# Uneven distribution of nucleoside transporters and intracellular enzymatic degradation prevent transport of intact [^14^C] adenosine across the sheep choroid plexus epithelium as a monolayer in primary culture

**DOI:** 10.1186/1743-8454-3-4

**Published:** 2006-03-29

**Authors:** Zoran B Redzic, Aleksandra J Isakovic, Sonja T Misirlic Dencic, Dusan Popadic, Malcolm B Segal

**Affiliations:** 1 School of Biomedical Sciences, King's College London, London, UK; 2 Department of Biochemistry, School of Medicine, Belgrade, Serbia & Montenegro; 3 Department of Microbiology, School of Medicine, Belgrade, Serbia & Montenegro; 4 Department of Physiology, Faculty of Medicine, Kuwait, Kuwait

## Abstract

**Background:**

Efflux transport of adenosine across the choroid plexus (CP) epithelium might contribute to the homeostasis of this neuromodulator in the extracellular fluids of the brain. The aim of this study was to explore adenosine transport across sheep CP epithelial cell monolayers in primary culture.

**Methods:**

To explore transport of adenosine across the CP epithelium, we have developed **a **method for primary culture of the sheep choroid plexus epithelial cells (CPEC) on plastic permeable supports and analysed [^14^C] adenosine transport across this cellular layer, [^14^C] adenosine metabolism inside the cells, and cellular uptake of [^14^C] adenosine from either of the chambers. The primary cell culture consisted of an enriched epithelial cell fraction from the sheep fourth ventricle CP and was grown on laminin-precoated filter inserts.

**Results and conclusion:**

CPEC grew as monolayers forming typical polygonal islands, reaching optical confluence on the third day after the seeding. Transepithelial electrical resistance increased over the time after seeding up to 85 ± 9 Ω cm^2 ^at day 8, while permeability towards [^14^C] sucrose, a marker of paracellular diffusion, simultaneously decreased. These cells expressed some features typical of the CPEC *in situ*, including three nucleoside transporters at the transcript level that normally mediate adenosine transport across cellular membranes. The estimated permeability of these monolayers towards [^14^C] adenosine was low and the same order of magnitude as for the markers of paracellular diffusion.

However, inhibition of the intracellular enzymes, adenosine kinase and adenosine deaminase, led to a significant increase in transcellular permeability, indicating that intracellular phosphorylation into nucleotides might be a reason for the low transcellular permeability. HPLC analysis with simultaneous detection of radioactivity revealed that [^14^C] radioactivity which appeared in the acceptor chamber after the incubation of CPEC monolayers with [^14^C] adenosine in the donor chamber was mostly present as [^14^C] hypoxanthine, a product of adenosine metabolic degradation. Therefore, it appears that CPEC in primary cultures act as an enzymatic barrier towards adenosine. Cellular uptake studies revealed that concentrative uptake of [^14^C] adenosine was confined only to the side of these cells facing the upper or apical chamber, indicating uneven distribution of nucleoside transporters.

## Background

Adenosine plays a general role in cellular metabolism, but within the CNS it has additional important effects as a neuromodulator. The net effect of adenosine in the CNS, although subject to complex regulation, depends also upon its concentration in the brain interstitial fluid (ISF). Depending on the class of receptors activated, adenosine can produce hyperpolarisation of neurons, inhibition of glutamate release from neurons and reduction of glutamate receptor activity [1] generating neuroprotective effects or contrasting neurotoxic effects [2].

Analysis of the brain ISF samples obtained by microdialysis has suggested that the concentration of adenosine in the brain ISF under normal resting conditions is low and remains within a narrow range, probably 120–210 nM [3]. Although some adenosine in the brain may be produced intracellularly through the action of soluble 5'-nucleotidases [4] and pass across the plasma membrane into the extracellular space *via *nucleoside transporters, the major source of adenosine in the brain ISF is extracellular production, mediated by the action of ecto-5'-nucleotidases on ATP [5]. It appears that the main homeostatic mechanism that opposes any increase in adenosine concentration in the ISF is cellular uptake into neurons/glia [6].

Efflux transport from the brain into the blood and/or removal into the cerebrospinal fluid (CSF) by the bulk flow of the brain ISF could be considered as additional pathways for removing adenosine from the brain ISF. Our recent studies in rat revealed that efflux transport through the blood brain barrier (BBB) makes a negligible contribution to adenosine removal from the ISF when compared to the cellular uptake [7]. However, when adenosine in the ISF increases, such as in a mismatch between energy demands and supply, the glial/neuronal transport system could become saturated, in this case removal by the brain endothelium may play a more significant role and account for ~25% of clearance [7].

Experimental evidence suggests that there is a slow current of ISF bulk flow towards the CSF (for the review see [8]), which might represent an additional route in the elimination of metabolites from the brain ISF. Once they reach the CSF, these molecules can then either enter the systemic circulation or the lymph by CSF bulk flow or they can be removed from the ventricular CSF into the blood by efflux transport across the epithelium of the four choroid plexuses (CPs), which form the blood-cerebrospinal fluid barrier (BCSFB) *in vivo*. Using the HPLC-fluorometric analysis, we have estimated that a concentration gradient from the CSF towards plasma exists in rat [7].

However, transport across the BCSFB depends not only on the concentration gradient of solutes but also on the transport properties of the CP epithelium. Our previous studies on the isolated CP of sheep perfused *in situ *have revealed that the basolateral membrane of the CP epithelium is very permeable to adenosine and guanosine but much less permeable towards pyrimidines [9]. However, this technique could only provide data regarding the transport properties of basolateral membrane of the CP epithelium, which faces the CP ISF *in situ*; no data exist regarding the transport across the apical (CSF-facing) membrane or transcellular transport of adenosine across the CP epithelial cells (CPEC) or the distribution of nucleoside transporters in the CPEC.

To explore transport of adenosine across the CP epithelium, we have developed primary culture of the sheep CPEC on plastic permeable supports and then analysed [^14^C] adenosine transport across this cellular layer, [^14^C] adenosine metabolism inside the cells, and cellular uptake of [^14^C] adenosine from either of the chambers. The results showed that the transport of [^14^C] adenosine across the CPEC monolayer was very low, which was probably a consequence of rapid intracellular trapping of this nucleoside by phosphorylation into nucleotides. Moreover, the radioactivity, which appeared in the acceptor chamber after the incubation of CPEC monolayers with [^14^C] adenosine in donor chamber, mainly represented [^14^C] hypoxanthine, a product of adenosine metabolic degradation. Therefore, it appears that the BCSFB in sheep represents not only a physical, but also an "enzymatic" barrier for adenosine. Also, uptake studies showed that the distribution of nucleoside transporters in these cells was uneven, with concentrative adenosine uptake confined only to the side that faced the upper or apical chamber, suggesting that the BCSFB might play a role in the adenosine efflux from the CSF.

## Methods

### Animal surgery and sheep choroid plexus epithelial cell harvesting

Animal care and procedures have been conducted according to the UK Home Office Animal Act (Scientific procedures), 1986 (Schedule 1). Adult sheep weighting 30–35 kg were anaesthetised with thiopentone sodium 25 mg/kg i.v. They also received heparin (150 IU/kg i.v.). The animal was decapitated, the skull was opened, *dura mater *dissected and whole brain removed and soaked in the sterile PBS in a semi-sterile hood. Fourth ventricle (4 V) and lateral ventricles (LVs) were opened, the choroid plexuses removed and placed in a warm, sterile PBS (37°C, pH 7.25), and transferred to the sterile hood. All CPs were quickly chopped into 2–3 mm diameter pieces, washed 3–4 times with warm PBS, transferred into dispase solution (2 U/ml dispase, Invitrogen, in PBS) and incubated at 37°C for 30 min. CPs from LVs were kept and processed separately from the 4 V CP.

After the incubation, CP pieces were triturated gently through a 1 ml pipette tip for 1–2 min in order to release more CP cells from the tissue. This cell suspension was filtered through 0.1 mm nylon mesh; centrifuged 5 min at 250 × g and the supernatant containing mostly single non-epithelial cells was discarded. The residual pellet consisted of large clusters of epithelial cells and will be referred to as an enriched epithelial cells fraction (EECF). In some cases EECF was used for PCR analysis. Otherwise, this fraction was re-suspended in the PBS containing 12.5 μg/ml DNase I (Roche Diagnostics) and gently triturated through a 0.2 ml pipette tip for 1–2 min in order to disperse large clumps of epithelial cells, then filtered through 0.04 mm nylon mesh and centrifuged again for 5 min at 450 × g. The supernatant was discarded and the pellet re-suspended in the final cell culture medium. This medium consisted of Ham's F-12 and DMEM (1:1) supplemented with 10% (v/v) foetal calf serum (FCS), 4 mM glutamine, 50 μg/ml gentamicin, 5 μg/ml insulin, 5 μg/ml transferrin, 5 ng/ml Na^+^-selenite, 10 ng/ml epidermal growth factor, 2 μg/ml hydrocortisone, 5 ng/ml basic fibroblast growth factor and essential fatty acids. The medium was prepared prior to the experiment, pH adjusted to 7.25, and then incubated (37°C, 5% CO_2 _in air) for 1 h before use.

Filter inserts (12 mm diameter, 1 cm^2 ^surface, 0.4 μm pore size; Costar Plastics) were pre-coated on the upper side with 8–10 μg/cm^2^of mouse laminin (Sigma), as described by the manufacturer. The cells were seeded on these inserts at a density of 3–4 × 10^5 ^cells/cm^2 ^and then plates with the inserts were left undisturbed in the incubator at 37°C and 5% CO_2 _in humid air for 48 h. After that time the inserts were washed twice with warm Dulbecco's PBS (DPBS, pH 7.3, with Ca^++ ^and Mg^++^), fresh pre-warmed final medium was added and the procedure was repeated each 48–72 h. In order to suppress fibroblast growth, 50 μg/ml of cys-hydroxy proline (Aldrich) was added to the final medium and cells were exposed to that medium for 36 h as soon as they reached optical confluence.

### Transepithelial electrical resistance

The transepithelial electrical resistance (TEER) was measured at a constant temperature in the World Precision Instruments (WPI, Sarasota, FL, USA) ENDHOLM chamber, containing DPBS (37°C, pH 7.3) with a WPI resistance meter and was expressed as Ω × cm^2^. Independent measurements were recorded three times for each filter and were then averaged. The TEER of laminin-coated, cell-free filters was measured as background and subtracted from values of the cell-seeded filters.

### Immunocytochemistry

Cellular monolayers were fixed for 20 min at 4°C in 4% paraformaldehyde, free aldehydes were then quenched with 75 mM NH_4_Cl and 20 mM glycine in PBS for 10 min, inserts were washed in PBS and permeabilized with methanol at -20°C, followed by a blocking step in 1% bovine serum albumin (BSA) in PBS for 30 min at room temperature. Cells were then incubated overnight at 4°C with one of the following primary antibodies: anti-8, 18 cytokeratin monoclonal mouse IgG (Sigma), working dilution (WD) 1:400, anti-occludin polyclonal rabbit IgG (Zymed, San Francisco, CA) WD 1:200, and anti-transthyretin goat polyclonal IgG, WD 1:400. In all cases controls, not shown in the results section, were run with isotype IgGs (mouse from Serotec, Oxford, and rabbit from Zymed). The inserts were then washed four times with 0.1% BSA in PBS and incubated for 1 h at room temperature with one of the following secondary antibodies: FITC-conjugated anti-mouse IgG (1:50; Jackson, West Grove, PA), FITC-conjugated anti rabbit IgG (1:100, Molecular Probes, USA) or FITC conjugated anti-goat IgG (1:100, Molecular Probes, USA). After washing, inserts were cut out from the plastic support, mounted on glass slides (with the cells facing the slide surface) and covered with cover slips. The preparations were examined on a Nikon fluorescence microscope.

### Transmission electron microscopy

Samples for electron microscopy were prepared on inserts with the cells cultured for 8d. Cells were fixed in 2% glutaraldehyde in 0.12 M sodium cacodylate buffer and 1 mM CaCl_2_, pH 7.4, for 30 min at 37°C. All subsequent steps were performed at 4°C. After washing, the samples were post-fixed with 1% osmium tetroxide-1.5% potassium ferricyanide for 30 min, washed again and stained in 1.2% uranyl acetate for 20 min. Tissues were dehydrated in graded alcohol and resin-embedded. Adjacent silver-to-pale gold ultrathin sections (~80–90 nm) were cut with a diamond knife and picked up on single-slot nickel grids. Grids were stained with uranyl acetate and lead citrate and examined using a Toshiba 1200 EX microscope.

### Scanning electron microscopy

The cells on the inserts were fixed for scanning electron microscopy (SEM) 8–9 days after seeding. The cells were fixed for 24 h in Karnowsky fixative at 4°C, postfixed in 4% OsO_4 _solution and then dehydrated in an ethanol series. The inserts were dried with CO_2 _at the critical point in a Blazers Union apparatus and then sputtering with gold was performed. Samples were examined in Zeiss SM 940A Scanning Electron Microscope.

### RT-PCR

In order to explore the amount of mRNA for three nucleoside transporters that mediate adenosine transport (which are: equilibrative nucleoside transporter 1 (ENT1), equilibrative nucleoside transporter 2 (ENT2) and concentrative nucleoside transporter 2 (CNT2)) and for transthyretin (TTR), total cellular RNA was isolated using the TRIzol (Gibco) from CPEC at different stages of culture (for nucleoside transporters and TTR), from freshly harvested CPs (only for TTR), from freshly isolated EECF for nucleoside transporters, from sheep heart (negative control for TTR), sheep liver (positive control for TTR) and whole brain homogenate (positive control for nucleoside transporters). RNA was reverse transcribed with Murine mammary lentivirus (MuMLV) reverse transcriptase (Invitrogen), according to the manufacturer's instructions using random hexamers (Pharmacia Biotech). The cDNA template was amplified using a Platinum Taq polymerase (Invitrogen).

There was no published sequence data available for nucleoside transporter genes in the sheep. Therefore, we have designed primers by comparing the published sequences of genes that encode these transporters in rat, to those in mouse and humans. Appropriate regions where the sequences were 100% preserved between these species were chosen: for ENT2, 445 bp product was generated with CCTACAGCACCCTCTTCCTC sense and GACAGGGTGACTGTGAAGA antisense corresponding to nucleotides 627–646 and 1071-1053 of the published rat ENT2 (AF015305.1) sequence; for ENT1 a 289 bp product was generated with CTCTCAGTGCCATCTTCAACA sense and TCCAACTTGGTCTCCTGCTC antisense corresponding to nucleotides 228–248 and 756-737 of the published rat ENT1 (NM_031684.1) sequence and for CNT2 305 bp product was generated with GCACTGGCCTTGTTTGTCA sense and TGGAGCAGGCAAAGAGGA antisense corresponding to nucleotides 555 – 573 and 819-802 of the published rat CNT2 (emb AL844566.8) sequence. The number of cycles for each gene was determined in preliminary experiments to ensure non-saturating PCR conditions and PCR reactions were then carried out employing the following thermal profile: denaturation 5 min at 95°C, then 10 cycles composed of 30 sec at 95°C, 1 min at 68°C with decrement of annealing/extension temperature of 1°C/cycle to achieve high specificity and then 25 cycles at 95°C for 30 sec, 58°C 1 min, and 74°C for 45 sec, and 7 min at 74°C.

To detect the presence of TTR mRNA a 436 bp product was generated using a primer GCTTCCTTCCGTCTGCTCC sense and CCTTGGGACTGCTGACAAG antisense primer corresponding to nucleotides 15–33 and 450-432, respectively of the published TTR sheep cDNA (emb X15576.1). The number of cycles was determined in preliminary experiments to ensure non-saturating PCR conditions and the PCR reaction was run employing the following profile: initial denaturation 5 min at 95°C followed by 28 cycles of denaturation at 95°C for 30 sec, annealing at 56°C for 30 sec and extension at 72°C for 40 sec, with final extension 7 min at 72°C.

The gene encoding glycolytic enzyme glyceraldehyde-3-phosphate dehydrogenase (GAPDH) was used as a housekeeping gene. GAPDH was co-amplified to normalize the amount of cDNA in different samples with CATGTTCCAGTATGATTCC sense and TTGCTGACAATCTTGAGG antisense primers corresponding to nucleotides 96–114 and 407-390 of the published sheep GAPDH sequence (AF035421.1) generating a 332 bp PCR product.

All PCR products were separated on 1.5% agarose gel containing ethidium bromide, bands were visualised under UV light and images were captured using a KODAK Gel Logic 200 camera. The density of bands was determined using NIH 1.61 PPC software, and relative expression was calculated as TTR or nucleoside transporter band density/GAPDH band density ratio.

### Uptake and permeability studies

All incubations were performed on a rotating platform at 37°C in an incubator (Stuart Scientific, Bath, UK) and on an orbital shaker (100 rpm) in order to avoid formation of an unstirred water layer. Since TEM and SEM studies have revealed that the side of the monolayer facing the upper chamber expressed some properties of the apical (CSF) side of CP epithelium *in situ*, the side of these cells facing this chamber will be referred to as apical, whereas the side of the CPEC facing the lower chamber will be referred to as basolateral. The chamber containing the uptake buffer with radioactive tracers will be referred as the donor chamber and the chamber containing buffer without radioactive tracers at the beginning of experiment, will be referred as the acceptor chamber. Permeability studies were performed using [^14^C] adenosine (specific activity 500–620 mCi/mMol, Amersham Biosciences), [^3^H] mannitol (74 Ci/mMole ; ICN) and [^14^C] sucrose (350 mCi/mMol, Amersham Biosciences). Uptake buffer contained (mM) 150 NaCl, 5.2 KCl, 2.2 CaCl_2_, 0.2 MgCl_2_, 6 NaHCO_3_, 3.5 glucose, 20 HEPES, FCS (1%) and was adjusted to pH 7.25. All unlabelled chemicals were purchased from Sigma.

### Transport with the apical chamber as the donor

Wells of a 12-well plate were filled with 1.2 ml of the uptake buffer. One insert covered by a 8d-old confluent monolayer of epithelial cells was set into one well, and 0.4 ml of the uptake buffer added to the apical chamber. The uptake buffer contained the following labelled molecules: a) 0.025 μCi of [^14^C] adenosine (final concentration 125 nM) and 0.1 μCi of [^3^H] mannitol, or b) 0.1 μCi of [^14^C] sucrose.

At regular intervals thereafter, the insert was transferred to another well to minimize the backflux of molecules from the acceptor to the donor chamber. Laminin-coated inserts without cells were also run in triplicate at the same time. Aliquots (50 μl) from the basolateral chamber as well as 20 μl aliquots from the donor chamber were collected after 1, 10, 20 and 30 min. The radioactivity in the samples was determined by liquid scintillation counting and counts per minute converted into disintegrations per minute (DPM) using an internal quench curve.

### Transport with the basolateral chamber as the donor

This transport was measured by the same method as explained above except that the uptake buffer with radioactivity (0.075 μCi of [^14^C] adenosine (final concentration 125 nM) and 0.3 μCi of [^3^H] mannitol) was placed in one well of a 12-well plate with the basolateral chamber as donor. An insert was lowered into that well and 0.4 ml of the uptake buffer without radioactivity added to apical chamber. An aliquot of 0.1 ml was removed from the apical chamber and replaced with fresh buffer at regular intervals in order to reduce the possible backflux from the acceptor chamber.

### Inhibition of adenosine metabolism

In some cases intracellular adenosine metabolism was reduced prior to transport study using a method explained elsewhere [10]. Briefly, 5 min prior to the study the cells were incubated in DMEM/F12 medium supplemented with 10% FCS and that medium contained an inhibitor of adenosine kinase (AK, E.C. 2.7.1.20) 5-iodo tubercidin (5-IT) 1 μM, an inhibitor of adenosine deaminase (ADA, E.C.3.5.4.4) erythro-9-(2-hydroxy-3-nonyl) adenosine (EHNA), 50 μM, and 10 mM of non-metabolized glucose analogue, 2-deoxy glucose. To determine if the exposure of confluent monolayers to these inhibitors affected the integrity of tight junction in that monolayer, the clearance of [^14^C] sucrose from the upper chamber as a donor was estimated after 10, 15, 20 and 30 min of incubation with CPEC monolayers.

### Calculation of [^14^C] adenosine flux across the monolayer

The flux of material across the monolayer was estimated as explained in Strazielle and Ghersi-Egea [14] with the theoretical background explained in [11]. Briefly, the reciprocals of the permeability-surface area (PS) product (in μl per min per filter) of the serially arranged layers composing the cell monolayer-laminin-filter system are additive and conform to the following equation:

1/PSt = 1/PSf + 1/Pse     (1)

where PSt and PSf are the PS products determined for filters with (t for total) and without (f for filter) epithelial cells, respectively, and PSe is the permeability-surface area product of the epithelial monolayer. The permeability quotient of the epithelial cells, Pe (cm/min) was obtained by dividing the PSe by the surface area.

### Cellular uptake of [^14^C] adenosine

Uptake studies were performed as described by Chishty et al. [10] and adenosine metabolism was reduced prior to these studies as described above. The uptake buffer was transferred to the inserts and the donor chamber contained 1405 ± 105 dpm/μl (mean ± SD, n = 15) of [^14^C] adenosine as a test molecule (final concentration 125 nM) and 1455 ± 122 dpm/μl (mean ± SEM, n = 15) of [^3^H] mannitol as a marker of test molecule trapping in the extracellular space. In some experiments NaCl and NaHCO_3 _in the uptake buffer were replaced by choline-Cl and choline-HCO_3_, respectively, in order to study the sodium-dependency of the adenosine uptake. In some cases, the uptake buffer contained the unlabelled nucleoside analogue nitrobenzylthioinosine (NBTI) in order to study its effect on adenosine uptake.

#### Uptake from the upper (apical) chamber

Wells of a 12-well plate were filled with 1.2 ml of uptake buffer and an insert covered by a confluent monolayer of CPEC set into each well. Then 0.4 ml of the same solution containing radioactive tracers was added to the apical chamber. At regular intervals (2.5 min) uptake buffer from the basolateral chamber was replaced with fresh uptake buffer. At the end of the experiment, which was 5 or 10 min after the initiation of uptake, the insert with the cells was removed from the well and washed twice with the ice-cold PBS. Cells were lysed using 0.1 ml/cm^2 ^of 2% Triton X-100 and the cell lysate was collected.

#### Uptake from the lower (basolateral) chamber

Uptake buffer containing radioactive tracers, was placed in one well of a 12-well and an insert containing 0.4 ml of the same buffer without radioactivity was inserted into the well. At regular intervals (2.5 min) uptake buffer from the upper chamber was replaced with fresh uptake buffer. The experiments were terminated as explained above.

#### Calculations

The results were expressed as dpm/mg protein. Protein was determined according to the Bradford assay and found to be 1.66 ± 0.2 mg/cm^2 ^(mean ± SD, n = 24). In order to correct for the amount of test molecule remaining in the extracellular space, dpm/mg protein of [^3^H] was subtracted from dpm/mg protein of [^14^C].

### The HPLC analysis with simultaneous scintillation counting

These experiments aimed to analyse if any metabolic transformation of adenosine takes place in the CPEC. Since the [^14^C] adenosine contains [^14^C] atom within the purine ring, the logic of experiments was that if metabolic transformation of [^14^C] adenosine into occurred in the CPEC, then that radioactive carbon atom will remain in the purine ring of metabolic product (i.e. nucleobase). These [^14^C] nucleobases would leave the cell and appear in the uptake buffer; since the retention times for nucleobases are rather different to the retention time for adenosine, the peak elution of radioactivity attached to nucleobases will appear at different time intervals to the peak elution of radioactivity attached to adenosine.

Therefore, the uptake buffer which contained 0.025 μCi of [^14^C] adenosine (final concentration 125 nM) was placed in one well of 12-well plate (basolateral chamber); then an insert with 8d-old confluent monolayers of the CPEC with the medium without radioactivity in the apical chamber was lowered into that well, the plate was placed on the rocking platform and 10 min after the beginning of the incubation a sample was collected from the apical chamber. Proteins in all samples were precipitated using methanol, samples were then centrifuged for 5 min at 12,000 g, at 4°C and 0.15 ml of supernatant was collected and stored in liquid N_2_.

HPLC analysis was performed on a JASCO HPLC system (JASCO, Great Dunmow, Essex, UK) with Packard radioactive detector (Packard, Pangbourne, UK). Samples were eluted over 20 min from the Intensil 51 ODS-2 (150 mm × 4.6 mm) column (GL Sciences, Tokyo, Japan) using a non-linear gradient of two buffers as reported previously [12]. The flow rate was 1.5 ml/min and the sample volume was adjusted by auto-sampler. Detection was performed at 254 nm on the UV and diode array detector. Peaks were detected and measured with a Jasco Control Software Workstation using external standards. Following HPLC analysis, the column outflow continued to a radioactive detector, where it was mixed with scintillation fluid (Ultima Flo M; Packard) and passed through a 0.5 ml flow cell for real-time radioactivity analysis.

## Results and discussion

### Sheep choroid plexus epithelial cells in primary culture form monolayers on laminin-coated inserts

Separate primary cultures of epithelial cells were initiated from CPs harvested from LV and 4 V. We found that a significantly smaller number of epithelial cells could be harvested from LV CPs for the same time of incubation in dispase solution (7.4 ± 1.3 × 10^5 ^cells/100 mg wet weight) than from 4 V CP (10.4 ± 1.5 × 10^5 ^cells/100 mg wet weight), while the plating efficiencies of the cells from these two origins were alike (mean ± SD: 9.1 ± 2.1%, n = 7 and 11.7 ± 3.5%, n = 12 for LV CPs and 4 V CP, respectively). However, fibroblast contamination appeared to be a major problem if LV CPs were used. For these reasons the data presented here represent results obtained on the epithelial cells from 4 V CP.

Various enzymatic digestions were tested using dispase or other proteolytic enzymes (0.25% trypsin and 1% pronase). Although significantly more cells were harvested after the digestion in trypsin or pronase than after the digestion in dispase (data not shown), the plating efficiency was lower and fibroblast contamination was more severe, so use of these enzymes was abandoned.

**Figure 1:**
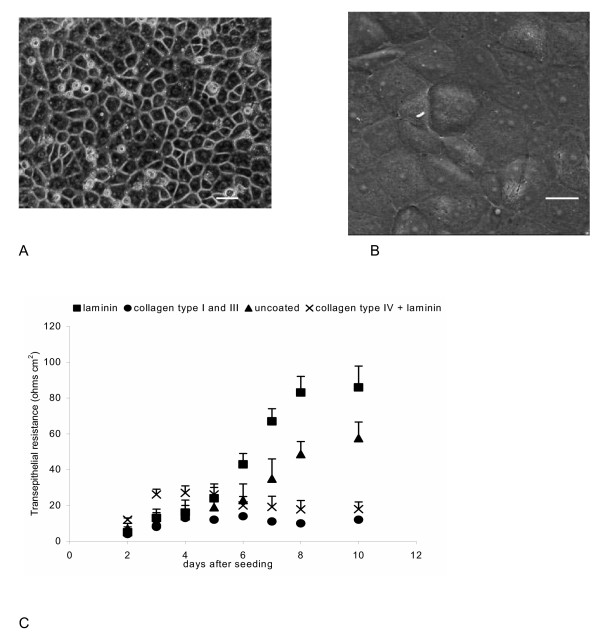
Morphology and phenotype of primary culture of sheep CPEC. (A) Phase-contrast micrographs of 8d-old CPE cells cultured on laminin-coated filters shows a typical cobblestone arrangement of polygonal cells (scale bar 20 μm); (B) Scanning electron microscopy shows a confluent 8d-old monolayer of CPEC, with a prominent nuclei (scale bar 10 μm). (C) Changes with time of TEER in CPEC monolayers seeded on inserts pre-coated with various basal lamina components or on uncoated inserts. Note the marked increase in TEER between days 3 and 8 across the CPEC monolayers seeded on inserts pre-coated with laminin and across CPEC monolayers seeded on uncoated inserts. Values are expressed as mean ± SEM from 3–4 different filters and were corrected for the mean TEER from three laminin-coated filters without cells.

Four different substrates were tested to reconstitute a basement membrane: a combination of collagen I and III, collagen IV, laminin, and a combination of laminin and collagen IV; in all cases pre-coating was performed following the instructions of the manufacturer. Cell attachment on laminin-coated insets occurred within several hours after plating and was completed within 36 h, so the inserts were washed and the medium changed for the first time after 48 h. The cells grew as densely packed small polygonal cells and produced a monolayer displaying a typical cobblestone-like appearance as shown by phase contrast microscopy and SEM (Figs. 1A,B) and formed **a **hydrodynamic barrier at the day 3 after the seeding, which was accompanied by an increase in TEER (Fig. 1C). After seeding the cells on plastic which was coated with basal lamina components other than laminin, the apparent plating efficiency was lower and the fibroblast contamination more severe (in the case of collagen I/III) or some cells detached from the monolayer 3–4 days after seeding (in the case of collagen IV).

Moreover, TEER across these monolayers remained several fold lower than across the monolayers on laminin-coated inserts (Fig. 1C). Therefore, collagens were abandoned and all results presented below were obtained on CPEC monolayers grown on laminin-coated inserts.

**Figure 2:**
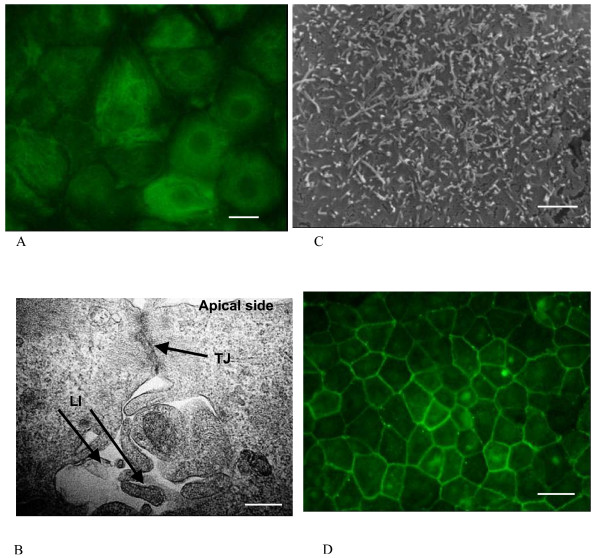
(A) The immunofluorescence assay using anti-cytokeratin antibodies showing a positive staining of the 8d-old monolayer of the CPEC (scale bar 10 μm). (B) Transmission electron micrographs of cultured CPE cells demonstrating the ultrastructural features of a polarized epithelial cell monolayer such as a tightly apposed lateral membrane with complex apical junctions organized as a tight junction (TJ) association and complex lateral interdigitations (LI) at the basolateral side (scale bar 0.01 μm, magnification 125.000 ×). (C) Scanning electron micrograph showing a number of processes on the CPEC side which faced apical (upper) chamber (scale bar 2 μm, magnification 6000 ×). (**D**) Eight-day-old CPE cells grown on laminin-coated filters were stained with primary antibodies against occludin and then with FITC conjugated secondary antibodies. A continuous circumferential distribution of fluorescence consistent with the establishment of TJs in CPEC monolayers is shown. Scale bar 20 μm.

The epithelial phenotype of these cells was further demonstrated by positive staining with a mixture of anti-cytokeratin monoclonal antibodies (Fig. 2A) that recognize epithelial types of keratin (types 8, 18 and 19). This is consistent with the finding that choroid plexus epithelium expresses these keratins *in vivo *[13]. Variation in fluorescence intensity between cells was also obvious (Fig. 2A) and this probably reflects a differential expression of the various cytokeratin subtypes, which was also shown in primary culture of rat CPEC [14].

Transmission (TEM) and scanning electron microscopy (SEM) were performed to determine if the cells in culture displayed some of the ultrastructural features characteristic of the CP epithelium *in situ*. TEM examination revealed that intercellular junctional complexes were present as electron dense areas close to the apical end of the lateral faces of adjacent cells (Fig. 2B). Another important feature of CP epithelia monolayer *in vitro *was that the lateral cell surfaces extended beneath these junctions in complex interdigitations forming complex infoldings (Fig. 2B), which is one of the important features of CP epithelia *in situ *[15]. SEM revealed the presence of uneven apical surface consisting of numerous cellular micro processes (Fig. 2C).

### CPEC monolayers develop functional barrier properties

The tight junctions (TJs) are complex protein structures and include transmembrane proteins interacting with cytoplasmic proteins and the cytoskeleton. The transmembrane protein, occludin, is exclusively located at TJs in epithelia and has a crucial role in regulation of TJ permeability [16]. Occludin distribution was therefore investigated in 8d-old monolayers of CPEC. An anti-occludin antibody stained a junctional ring around the cells without discontinuities (Fig. 2D). This confirms the establishment of TJs in CP primary cultures, previously suggested by the TEM examination.

**Figure 3:**
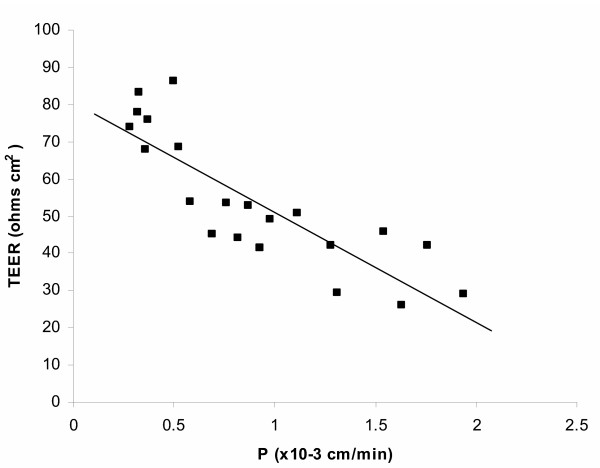
A plot of the TEER across CPEC monolayers against the permeability to [^14^C] sucrose. All 21 measurements were made on confluent monolayers from day 5 to day 8 after the seeding. TEER across laminin-coated inserts, which were kept under the same conditions, was subtracted as the background. The points revealed a strong linear reverse proportion between these two parameters, with Pearson's quotient -0.847.

In addition to that, the *in vitro *establishment of TJs was also examined by functional studies: monitoring TEER and measurement of paracellular flux of [^14^C] sucrose, a marker of paracellular diffusion, from the apical chamber as a donor. Although optical confluence appeared at day 2–3 after seeding, TEER significantly increased between day 4 and 6 (Fig. 1C) and reached 85 ± 9 Ω cm^2 ^at day 8; it did not show a further significant increase (it was 91 ± 12 Ω cm^2 ^at day 10). Paracellular permeability, estimated by measuring the permeability of CPEC monolayers towards [^14^C] sucrose, significantly decreased over time after seeding. Fig. 3 shows the plot of 21 TEER values 5–8 days after seeding against permeability for [^14^C] sucrose of the same monolayers. The Pearson quotient of these data points was -0.847, which indicated strong negative correlation between these TEER and permeability towards [^14^C] sucrose. It should be noted that, although CPE cells are sealed *in vivo *by a continuous belt of TJs and strongly impede the passive diffusion of polar compounds, this epithelium is not as tight as the cerebral capillary endothelium forming the blood-brain barrier and has been classified, based on functional grounds, in the category of 'leaky' epithelia [17].

To further investigate formation of Tjs and their influence on resistance and transport properties across the monolayers, TEER was measured in six 8d-old inserts and it was found to be 87 ± 7 Ω cm^2^. These inserts were then incubated in Hanks balanced salt solution without Ca^++ ^and Mg^++ ^(Gibco), containing 10% BSA for 1 h; after that period TEER decreased to a value of 4.2 ± 3.7 Ω cm^2^. This finding is consistent with the role of Ca^++ ^and Mg^++ ^in the functional structure of TJs.

### CPEC monolayers expressed transthyretin at the transcript and protein level

**Figure 4:**
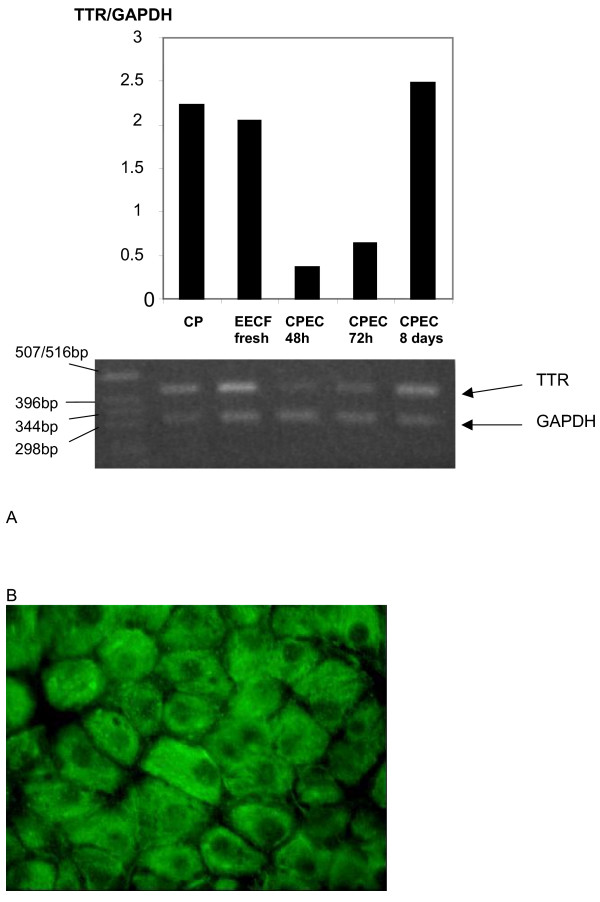
(A). Expression of transthyretin in CPEC cells at the transcript level. Total mRNA was isolated from sheep 4 V whole CP, EECF and CPEC in primary culture at 48 h, 72 h and 8d after seeding. The 436-bp-long and 332-bp-long fragments amplified using oligonucleotides specific for transthyretin and GAPDH, respectively, were visible in samples from both fresh tissue and cellular cultures at various periods after seeding. From these gels the expression of TTR was estimated relative to GAPDH and the values presented in the bar graphs. They show that TTR mRNA expression is similar to that of fresh tissue in 8d-old cultured cells. Sheep liver and heart homogenate were processed in parallel as positive and negative controls, respectively (not shown). Far left lane contains DNA molecular weight markers. (B) Expression of transthyretin in 8d-old monolayers of CPEC cells at the protein level. CPEC were treated with goat anti-transthyretin polyclonal IgG (1:400) and then with mouse anti-goat IgG. There was intensive fluorescence in the cytoplasm, while the nuclei were not stained. Scale bar 10 μm.

The majority of protein transthyretin (TTR) is synthesized in the liver, while in the brain TTR is exclusively synthesized and secreted by the CPEC [18] and could be regarded as tissue-specific and a marker of CP epithelia differentiation [19]. Therefore, we measured the amount of TTR mRNA, relative to the amount of mRNA for the housekeeping gene GAPDH, in the cells in primary culture at different times after seeding and in the freshly harvested sheep CP. At 48 h and 72 h after the seeding the relative expression of TTR mRNA in attached cells was much lower than in the freshly isolated EECF (Fig. 4A). However, the amount of TTR mRNA apparently increased over time since the relative expression in the 8d-old monolayers did not differ from the relative expression in freshly isolated CP cells. The presence of TTR protein in 8d-old CPEC monolayers was also confirmed at the protein level, using anti-TTR IgG antibodies (goat origin). These monolayers showed intensive staining suggesting presence of this protein in cytoplasm (Fig. 4B).

### Transport studies

#### Eight days old CPEC monolayers expressed ENT1, ENT2 and CNT2 at the transcript level

On the basis of functional activity, nucleoside transport processes in mammalian cells have been categorized into two groups: the first group consists of Na^+^-independent (equilibrative) nucleoside transport processes and these processes are inhibited by synthetic nucleoside analogue nitrobenzylthioinosine (NBTI). A second group consists of Na^+^-dependent nucleoside transport processes; they have the ability to concentrate nucleosides intracellularly against a concentration gradient and some of them are resistant to inhibition by NBTI (for the early review see [20]). Equilibrative transport is found in most mammalian cells, whereas concentrative transport is generally limited to specialized cell types, usually epithelia [21]. The cDNAs encoding membrane proteins with nucleoside transport activity have been isolated and functionally expressed in oocytes of Xenopus laevis [22,23]. These transporters comprise two protein families: one family mediates Na^+^-independent nucleoside transport and consists of at least four equilibrative nucleoside transporters (ENT) 1–4 [24] and the second family mediates Na^+^-dependent nucleoside transport and consists of at least three concentrative nucleoside transporters (CNT) 1–3 [25].

**Figure 5:**
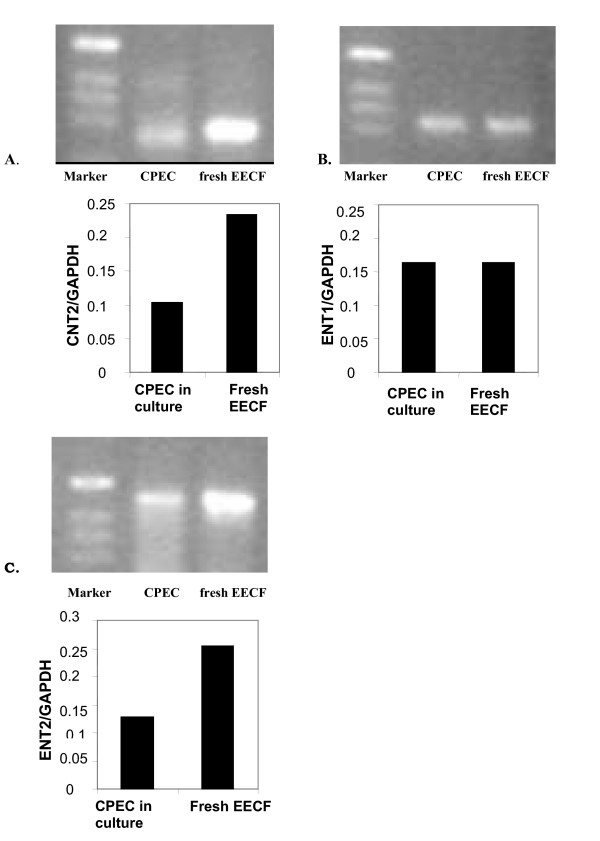
The expression of (A) CNT2, (B) ENT1, and (C) ENT2 at the transcript level in the 8d-old CPEC in primary culture and in the fresh EECF. All gels revealed bands corresponding to these nucleoside transporters in both fresh EECF and CPEC in primary culture. They were also present in samples from whole brain homogenate (positive control, not shown in the figure). The amount of mRNA for these proteins was expressed relative to the amount of mRNA for the housekeeping protein GAPDH and the mean values are presented as bars. The apparent amount of mRNA for GPADH did not differ between fresh EECF samples and samples from CPEC in primary culture. The relative expression of mRNA for ENT1 was the same in CPEC in primary culture as in EECF; however, relative expressions of mRNA for two other proteins were 40–50% lower in CPEC than in EECF.

The affinity of these transporters for various nucleosides differs [26]. Three nucleoside transporters mainly mediate transport of adenosine across the plasma membrane in most cells: the equilibrative transporters ENT1 and ENT2 and the concentrative transporter CNT2 [26]. Since no published data existed regarding the expression of these three transporters in sheep CPEC, we tested their expression at the transcript level in the fresh EECF and in the CPEC in primary culture. Whole brain homogenates were analysed by PCR at the same time as positive controls. These gels revealed bands of the expected sizes for all three nucleoside transporters (data not shown). Bands for all three transporters are clearly visible in the samples from 8d-old CPEC monolayers and in the samples of fresh EECF (Figs. 5A,B,C). Amounts of ENT1, ENT2 and CNT2 mRNA were estimated relative to the amount of mRNA for the housekeeping gene GAPDH (Figs. 5A,B,C). The amount of GAPDH mRNA did not differ between EECF and CPEC (data not shown). The relative amount of mRNA encoding ENT1 in CPEC in culture was similar to the freshly isolated EECF, while expressions of mRNA for ENT2 and CNT2 were 40–50% lower in CPEC in primary culture than in EECF. The assessment of the expression of these transporters at the protein level could not be performed since no antibodies are available against these proteins in sheep and anti-rat nucleoside transporters antibodies do not cross-react with sheep. Our recent study on rat CPEC in primary culture revealed that these cells also express mRNA for rENT1, rENT2, rCNT2 and to lower extend rCNT3, while the band for rCNT1 was nearly invisible [27].

#### Permeability of the CPEC monolayers for [^14^C] adenosine was low and appeared to be a consequence of intracellular trapping

Transepithelial permeability towards [^14^C] sucrose reflects the paracellular pathway and in 8d-old monolayers it appeared to be sufficiently limited to allow precise measurement of transcellular permeability across the monolayer (Fig 3).

**Figure 6:**
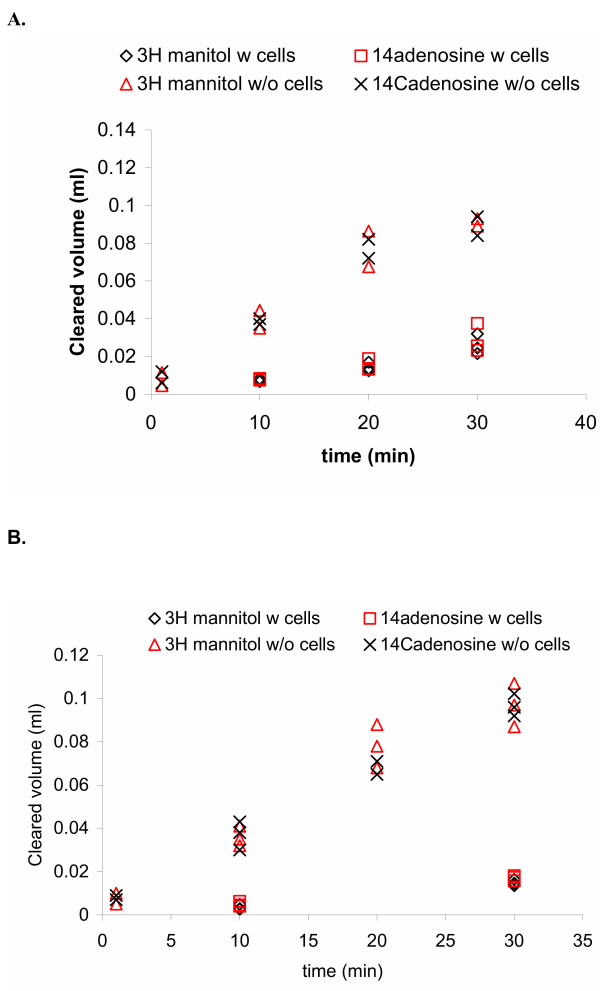
Clearance of [^14^C] adenosine and [^3^H] mannitol across the 8d-old CPEC monolayers. (A) The donor was the upper chamber. (B) The donor was the lower chamber. Experiments were performed on CPEC monolayers from at least 2 animals. The concentration of [^14^C] adenosine was 125 nM, which represented about 5% of Km of [^3^H] adenosine uptake by the basolateral side of sheep CPEC *in situ *(I. Markovic, PhD Thesis, University of Belgrade, 1998). The clearance was linear; however, the values obtained were quite close to the values of [^3^H] mannitol clearance. Using these data points the permeability of CPEC monolayers for [^14^C] adenosine was calculated and these values are presented in Table 1.

The values of clearances of [^14^C] adenosine and [^3^H] mannitol from the donor chamber were calculated using the calculations explained elsewhere [14] and are presented in Fig. 6A (donor was the upper chamber) and 6B (donor was the lower chamber). Using these data-points, the permeability of the monolayer towards this molecule was calculated using equation 1 and these values are presented in Table 1. Permeability of the monolayers for adenosine was low in both directions and within the same order as the permeability for markers of paracellular diffusion, mannitol or sucrose.

**Table 1:** The permeability values (Pe - x 10^-3^cm/min) of laminin-coated inserts without cells (w/o cells), laminin coated inserts with 8d-old CPEC monolayer (with cells control) and laminin coated inserts with 8d-old CPEC monolayers which were pre-treated to reduce adenosine metabolism (with cells inhibition). [^14^C] adenosine was used as a test molecule and [^3^H] mannitol as an inert reference of the paracellular diffusion. From these values the permeability of CPEC monolayers (P_cells_) was calculated and then permeability for mannitol subtracted from the permeability for adenosine to estimate permeability of cellular monolayers for [^14^C] adenosine. All values are mean+/- SEM from 3–4 different inserts, which were produced from at least two animals.

	Upper chamber as the donor		Lower chamber as the donor	
	Adenosine	Mannitol	Adenosine	Mannitol
W/o cells	3.26 ± 0.76	3.40 ± 0.068	3.09 ± 0.41	3.32 ± 0.22
With cells control	0.64 ± 0.17	0.35 ± 0.11	0.73 ± 0.21	0.55 ± 0.08
With cells inhibition	1.50 ± 0.15	0.39 ± 0.10	1.35 ± 0.16	0.44 ± 0.08
Pe_cells _control	0.79 ± 0.05	0.39 ± 0.06	0.95 ± 0.11	0.64 ± 0.07
**Pe_ado_-Pe_mannitol _control**	**0.40 ± 0.03**		**0.31 ± 0.04**	
Pe_cells _inhibition	2.78 ± 0.14	0.44 ± 0.11	2.39 ± 0.17	0.67 ± 0.09
**Pe_ado_-Pe_mannitol _inhibition**	**2.34 ± 0.17**		**1.72 ± 0.14**	

In a number of mammalian cell types adenosine is rapidly metabolised in the cell *via *two different pathways: phosphorylation which is mediated mainly by the adenosine kinase (AK) and by degradation into nucleobases; the first reaction leading to degradation is mediated by ADA. To determine if the intracellular metabolism of adenosine might be a reason for low transepithelial permeability, intracellular metabolism of adenosine was inhibited prior to transport study in a separate set of experiments. Since the physical integrity of the monolayer is crucial for the accurate estimation of transcellular permeability, the effects of the reduction of adenosine metabolism on the paracellular permeability across these monolayers was also explored. The clearance of [^14^C] sucrose, a marker of a paracellular diffusion, across these monolayers after 10 and 15 min were 10 ± 4 μl and 16 ± 3 μl, respectively, which was not different from clearance across inserts which were not pre-treated to reduce metabolism (controls) (*P *> 0.05 for both by ANOVA). However, after 20 and 30 min of incubation the volumes of clearance were 28 ± 4 μl and 55 ± 8 μl, respectively, which was significantly higher than in controls (*P *< 0.05 and *P *< 0.01, respectively, by ANOVA). Therefore, the study of adenosine transport under these conditions was limited to 15 min.

The permeability values of CPEC monolayers for [^14^C] adenosine, estimated from the samples obtained after the inhibition of adenosine metabolism, are presented in Table 1: the apparent permeability has increased more than five-fold when compared to controls in both directions (*P *< 0.001 by ANOVA). These findings suggested that intracellular trapping of [^14^C] adenosine by phosphorylation into nucleotides and/or metabolic degradation into nucleobases, which show different transport kinetics to adenosine, might be the reason for the apparently low transcellular permeability of CPEC monolayers towards this nucleoside.

#### HPLC analysis with simultaneous scintillation counting revealed that the majority of [^14^C] radioactivity appeared as hypoxanthine in the acceptor chamber

**Figure 7:**
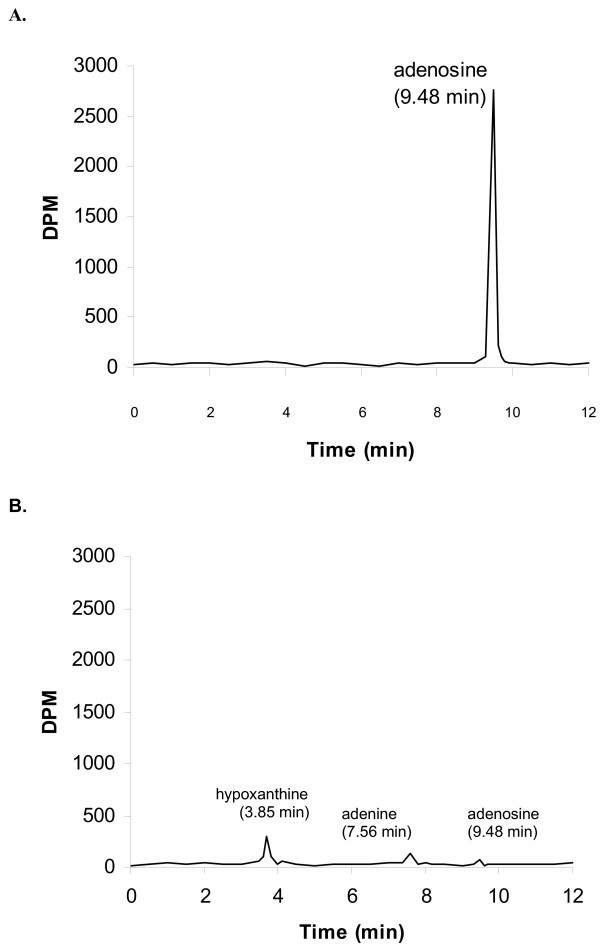
HPLC-radiodetector analysis of standard, which represented the uptake buffer from (A) the donor chamber containing [^14^C] adenosine prepared as described in Methods section and (B) of the uptake buffer from acceptor chamber collected after 10 min of incubation with CPEC monolayers. Y-axis shows DPM, and retention time in minutes is on the X-axis. Under these conditions, peak elution of radioactivity in the standard occurred at 9.28 min. However, a negligible amount of radioactivity was eluted at this time in the sample from the acceptor chamber (B), where about 2/3 DPM appeared in the hypoxanthine peak and the rest in the adenine peak. These peaks were identified by retention times and spectral analysis.

To further explore metabolism of [^14^C] adenosine within the CPEC in primary culture, we performed a transport study using only [^14^C] adenosine (without [^3^H] reference) and then analysed samples from the acceptor chamber by HPLC with the simultaneous detection of radioactivity. Typical plots of DPM in the eluted fluid versus retention time are shown in Figs. 7A and 7B. The chromatogram (7A) was obtained by analysis of the [^14^C] adenosine standard. In order to achieve the threshold of the detection by HPLC with the UV detection, 1 μMole of unlabelled adenosine was added to this standard prior to analysis. Under the chromatographic conditions employed, adenosine gave a retention time for UV absorbance of 9.22 min (chromatograms of the UV absorption are not shown) and this was accompanied by the peak elution of radioactivity at 9.48 min (Fig. 7A). Retention times of [^14^C] adenine and [^14^C] hypoxanthine standards (which were prepared in the same manner as the [^14^C] adenosine standard) were 3.64 and 7.35 min, respectively and the peak of elution of radioactivity occurred at 3.85 min and 7.56 min, respectively. The plot (Fig. 7B) was obtained from the sample collected from the acceptor chamber (in this case upper or apical chamber) after 10 min of incubation. HPLC analysis showed that a negligible amount of [^14^C] appeared within the adenosine peak, a minor part appeared within the adenine peak, while most of the remaining activity (>75%) appeared within the hypoxanthine peak. Our previous study, which was conducted on the isolated CP of the sheep perfused *in situ*, revealed that after the introduction of adenosine from the basolateral ('blood') side, mainly hypoxanthine appeared in the samples collected from the apical ('CSF') side. A similar finding was also revealed in the study on the efflux transport of [^14^C] adenosine across the rat BBB: after the intracerebral micro-injection of a bolus containing [^14^C] adenosine, the majority of [^14^C] radioactivity that appeared in the plasma collected from jugular veins was present as [^14^C] hypoxathine and [^14^C] adenine [7].

#### Cellular uptake studies revealed uneven distribution of concentrative nucleoside transport in CPEC

**Figure 8:**
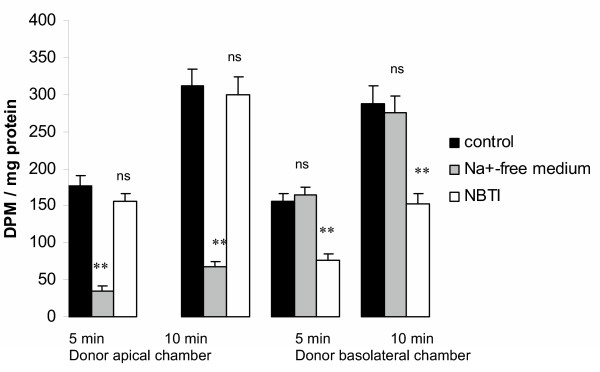
Uptake of [^14^C] adenosine into primary cultured sheep CPEC. The panel shows the cellular uptake from the apical (upper) chamber as a donor (left side) and from the basolateral (lower) chamber as a donor (right). Values shown represent uptake of adenosine, corrected for tracer trapped in the extracellular space, after 5 or 10 min of incubation in the uptake buffer containing [^14^C] adenosine and [^3^H] mannitol in the donor chamber. Data are shown for uptake under control conditions in Na^+^-containing medium (black bars), using Na^+^-free uptake buffer (grey bars), or in uptake buffer containing both Na^+ ^and 1 uM NBTI (open bars). All values are presented as mean ± SEM from three to five separate inserts obtained from at least two separate isolations. Statistical significance: n.s., *P *> 0.05 vs. control; **P < 0.01 vs. control by ANOVA.

Since rapid metabolic degradation of adenosine has been revealed, the distribution of nucleoside transporters at the CPEC in primary culture was probed by observing the effects of lack of Na^+ ^or exposure to NBTI on the uptake of [^14^C] adenosine into these cells, rather than on transcellular flux of [^14^C] radioactivity. The characteristics of [^14^C] adenosine uptake revealed a polarized distribution of nucleoside transport activities (Fig. 8). Replacement of Na^+ ^by choline produced a reduction in adenosine uptake across the apical membrane of the CPEC monolayer by approximately 2/3, but was without significant effect on uptake across the membrane facing the lower, basolateral chamber. These findings indicated that concentrative nucleoside transport occurs exclusively across the apical membrane. NBTI-insensitive, equilibrative transport was evident across both membrane domains but NBTI-sensitive equilibrative transport was detectable only across membranes facing the lower chamber. Polarization of membrane proteins between apical and basolateral membrane of CP epithelium is a feature involved in a number of the CP functions (for review see [28]) and a very similar pattern of distribution of ENTs and CNTs was revealed in rat CPEC in primary culture [27]. The pattern of distribution of nucleoside transporters revealed in this study, together with the rapid metabolism of adenosine inside CPEC, suggests that the CP epithelium might play roles in preventing influx of circulating adenosine into the CSF and in removing adenosine that has gained access to the CSF in the ventricles.

## Conclusion

Sheep CPEC in primary culture could be used as a productive experimental model in studies of transport at the BCSFB. Eight-day old monolayers of these cells expressed some feature typical of the CPEC *in situ *such as TJs between adjacent cells rich in occludin, lateral interdigitations and synthesis of the TTR. They developed relatively high TEER between days 4–8 after the seeding, which was accompanied by low paracellular permeability, a property that enabled the use of this model for studies of transcellular transport of adenosine. Transcellular permeability of these monolayers towards [^14^C] adenosine was surprisingly low and within the same order of magnitude as for inert markers of paracellular diffusion. However, inhibition of adenosine intracellular metabolism leaded to more than a five-fold increase in permeability for adenosine, indicating that intracellular phosphorylation of adenosine into nucleotides and/or its degradation into nucleobases which show different kinetic properties to adenosine might be the cause of low transcellular permeability. HPLC analysis with simultaneous detection of radioactivity revealed that [^14^C] radioactivity which appeared in the acceptor chamber after the incubation of CPEC monolayers with [^14^C] adenosine in the donor chamber, mostly was present as [^14^C] hypoxanthine, which indicated that sheep CPEC in primary culture also act as an enzymatic barrier towards adenosine. Cellular uptake studies revealed that concentrative uptake of [^14^C] adenosine was confined to the apical ('CSF') side of these cells, indicating uneven (polar) distribution of nucleoside transporters in sheep CPEC in primary culture.

## Abbreviations

ISF – interstitial fluid

CPEC – choroid plexus epithelial cells

EECF – enriched epithelial cell fraction

BCSFB – blood-cerebrospinal fluid barrier

SEM – scanning electron microscopy

TEM – transmission electron microscopy

ENT – equilibrative nucleoside transporter

CNT – concentrative nucleoside transporter

NBTI – nitrobenzylthioinosine

GAPDH – glyceraldehydes-3-phosphate dehydrogenase

AK – adenosine kinase

ADA – adenosine deaminase

## Competing interests

The author(s) declare that they have no competing interests.

## Authors' contributions

ZBR produced primary cultures of sheep CPEC, characterised it, performed all transport and HPLC studies and drafted the manuscript. AJI performed all immunocytochemical studies and contributed to some transport studies. STM contributed to transport studies. DP performed all PCR work. MBS supervised work throughout the study. All authors read and approved the final manuscript.
